# Physical activity predicts task‐related behaviour, affect and tiredness in the primary school classroom: A within‐person experiment

**DOI:** 10.1111/bjep.12523

**Published:** 2022-06-03

**Authors:** Christina Heemskerk, Steve Strand, Lars‐Erik Malmberg

**Affiliations:** ^1^ University of Bern Bern Switzerland; ^2^ University of Oxford Oxford UK

**Keywords:** affect, classroom behaviour, learning experiences, physical activity

## Abstract

**Aim:**

We investigated the dose–response relationship between acute physical activity (PA) intensity during physical education (PE) lessons (dose), and task behaviour and learning experiences in the classroom after PE (response), and mediation effects of acute PA on‐task behaviour via learning experiences.

**Method:**

A total of 78 children (*M*_age_ = 9.30 years; 43 females) took part. Participants reported learning experiences (tiredness, positive and negative affect) during one afternoon per week for 6 weeks. Their task behaviour was observed (on‐task, active off‐task and passive off‐task) during two classroom lessons. Between the classroom lessons, they took part in a PE lesson, with experimentally induced PA intensity (low, medium and high). Accelerometers were worn for 24 h leading up to and during every intervention afternoon. Participants completed self‐reports three times per classroom lesson, both before and after PE. Intra‐ and interindividual differences in PA, task behaviour and learning experiences were analysed with multilevel structural equation models.

**Results:**

Moderate PA directly increased on‐task behaviour and reduced passive off‐task behaviour, whereas light PA increased active off‐task behaviour and reduced on‐task behaviour. We found no direct effects of vigorous PA or mediated effects of any PA intensity on‐task‐related behaviour. However, a greater positive affect during PE indirectly led to more on‐task and less passive off‐task behaviour. Regularly active children reported less tiredness in the classroom.

**Conclusion:**

PE lessons can increase on‐task behaviour and reduce both passive and active off‐task behaviours. Positive affect and tiredness are indirectly involved in the impact of PA on task‐related behaviour. The greatest benefits were found for moderate PA and for PE lessons, which left children feeling positive. Moreover, regular participation in moderate‐to‐vigorous PA leads children to feel less tired during school lessons.

## INTRODUCTION

**FIGURE 1:**
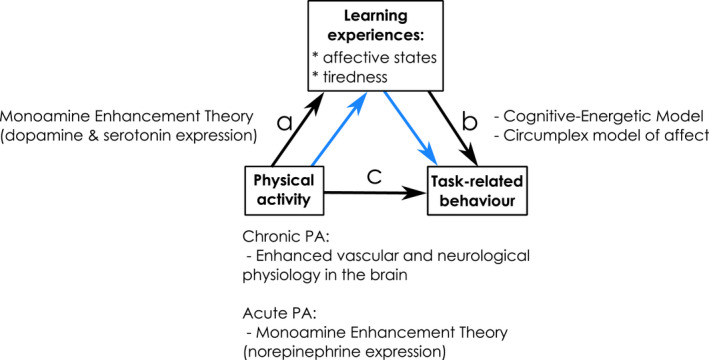
Proposed direct and mediated effects of physical activity and learning experiences on‐task‐related behaviour, and the underpinning theories. *Note*: A: Direct effect of physical activity on learning experiences; b: direct effect of learning experiences on task‐related behaviour; c: direct effect of physical activity on task‐related behaviour; a*b: mediated effect of physical activity on task‐related behaviour via learning experiences (blue arrows)

Children's positive learning experiences and on‐task behaviour at school are associated with academic success and positive outcomes beyond the school years (Fredricks et al., 2004; Roorda et al., 2011). Both habitual and experimentally induced physical activity (PA) have been found to have positive effects on task‐related behaviour and academic performance, and cognitive and mental well‐being in the longer term (Lubans et al., 2016). For PA interventions with primary school children, effect sizes of .26 (Fedewa & Ahn, 2011) and .27 (De Greeff et al., 2018) have been reported. Some dose–response effects have been found, indicating that more physically active children academically outperform less physically active children (Donnelly et al., 2016), but dose–response effects of habitual and acute PA on situational experiences of affect and tiredness are still inconclusive. Moreover, no consensus has been reached on the possible mediating effect of affect and tiredness in the relationship between PA and task‐related behaviour. In the present study, we go beyond previous studies on associations between subjective learning experiences, task behaviour and PA, by investigating both the direct dose–response effects of PA intensity during PE (the dose) on task‐related behaviour (the response), and mediated effects of PA via affect and tiredness, as illustrated in the schematic of proposed relationships in Figure 1.

### Physical activity and on‐task behaviour

There is increasing evidence that task‐related classroom behaviour, mostly operationalized as time‐on‐task, is improved by PA. Studies in both pre‐adolescents (e.g. De Greeff et al., 2016) and adolescents (e.g. Mavilidi et al., 2021) indicate that attention and on‐task behaviour are positively affected by PA. Although results are heterogeneous, the majority of studies investigating on‐task behaviour in primary school classrooms have found positive effects of PA on on‐task behaviour (De Greeff et al., 2018; Watson et al., 2017).

Improvements in on‐task behaviour after PA have been attributed to PA‐induced improvement of inhibition leading to better impulse control (e.g. Mahar, 2019; Vogan et al., 2018). This mechanism is based on the monoamine enhancement theory: PA causes stimulation of these brain tissues in the form of altered levels of certain neurotransmitters, including norepinephrine (Hötting et al., 2016; Meeusen, 2006). Norepinephrine is important for attention allocation, and when circulating levels are too low (or too high), the individual is more likely to be distracted (Unsworth & Robinson, 2017).

While experimental studies confirm such associations, we still know less about the processes in real‐time (Tetzlaff et al., 2021; Trevillion et al., 2022) and the acute effects of physical education (PE) lessons in school. For a long time, it was assumed that PA intensity (low, medium, high) had an inverted U relationship with academic outcomes, so that moderate PA (MPA) was most beneficial to cognitive performance and on‐task behaviour (Tomporowski, 2003). More recently, vigorous PA (VPA) has been shown to also be beneficial, with high‐intensity PA interventions yielding positive results for cognitive health (e.g. Mavilidi et al., 2021; Moreau et al., 2017), and on‐task behaviour specifically (Ma et al., 2014).

### Physical activity, and affect and tiredness

Another suggested mechanism for the positive effect of PA on on‐task behaviour is a mediated relationship via PA‐induced improvements in the affective state (Lubans et al., 2016; Mavilidi et al., 2021). Before exploring this mechanism further, it is important to mention that the terms ‘emotion’, ‘affect’ and ‘mood’ are often used interchangeably. We will differentiate between emotions, affect and mood as follows. Moods are different from emotions as they are longer‐lasting (hours or even days) than the transient nature of emotions (Tyng et al., 2017). Affect also differs from emotion. The circumplex model of affect places emotions in a circular space formed by two bipolar dimensions; valence (pleasant‐unpleasant) and arousal (energetic‐tired) (Russel, 1980). As several distinct emotions can be included in each quadrant of affect (Russel, 1980), affect is a broader concept than the more discrete term ‘emotion’.

The suggested mediation path from PA, via improved affect, to task‐related behaviour is a combination of two relationships, as reflected by the two blue arrows in Figure 1. In this section, we explore the mechanism by which PA leads to improved affect. Similar to the direct effect of PA on cognition, this is based on the monoamine enhancement theory. In this case, it concerns alterations to the neurotransmitters dopamine and serotonin, resulting in improved affect and mood, and feelings of being energetic (Meeusen, 2006). This combination of feelings of positivity and being energized bears a strong resemblance to the ‘positive activated’ quadrant of the circumplex model of affect (Russel, 1980).

In a recent systematic review, Bourke et al. (2021) reported that acute PA—and moderate‐to‐vigorous PA (MVPA) in particular—increases situation‐specific positive affect at the within‐person level in children and adolescents, whereas the findings for negative affect and mood were mixed. They suggest that, although no significant acute effects of PA on negative affect were found, it is possible that regular PA might lead to an accumulation of positive feelings and reduce negative affect in the longer term (Bourke et al., 2021).

In children and adults, the effect of PA on mood and affect appears to be most strongly related to MVPA and VPA. Strauss et al. (2001) found higher self‐esteem ratings in children participating in more VPA (*d* = .58), whilst Dunton et al. (2014) reported that, in children aged nine to thirteen, higher levels of MVPA were associated with higher ratings of positive affect and feeling energetic, and lower ratings of negative affect 30 min later.

As part of the self‐reports, we explicitly asked for a rating of ‘tiredness' as an indicator of how energetic the participants were feeling. The reasons for including tiredness as a separate construct are two‐fold. Firstly, despite tiredness representing a lack of arousal—one of the two dimensions of the circumplex model (Russel, 1980)—due to the absence of a positive or negative valence, tiredness is not in itself reflected in the four quadrants of the circumplex model, but rather is at the negative end of the arousal axis (Russel, 1980). And secondly, tiredness may not relate to PA intensity in the same way that affect does. Although the monoamine enhancement theory poses that PA leads to increased positivity and arousal, the effects may differ at various intensities of PA. As discussed previously, positive affect has been reported to be improved particularly by VPA and MVPA (Bourke et al., 2021; Strauss et al., 2001). However, PA at high‐intensity places a high energy demand on the body, depletes the body's energy stocks and leads to greater tiredness (Ament & Verkerke, 2009).

To summarize, based on the current literature, it appears emotions and affect respond favourably to MPA and VPA, whereas tiredness has a U‐shaped relationship with exercise intensity. This means that perceived tiredness is lower after exercising at moderate intensity, but greater after high‐intensity exercise. Considering the potentially different relationship PA has with affect and tiredness, both constructs were included in the study in their own right. We investigated the acute effects of PA intensity on self‐reported positive and negative affect and feelings of tiredness at the end of the PE lesson and during the subsequent classroom lesson.

### Affect and tiredness, and task‐related behaviour

The second element of the mediated relationship consists of the effect of affect and tiredness on task‐related behaviour. Affect is hypothesized to influence on‐task behaviour in two ways: through the arousal and through the valence (positive/negative) of the affect. Affective states—both positive and negative—can be a distraction from the task at hand. The inhibition of distractions and the targeted allocation of attention are prerequisites for on‐task behaviour in the classroom (Fredricks et al., 2004). Affective states are a source of task‐irrelevant thinking, thus influencing attention allocation (Pekrun et al., 2002). Both positive and negative affect can lead to a decrease in attention allocation, as both can drain attentional resources (Meinhardt & Pekrun, 2003).

Alongside valence, we must consider the effect of arousal on task focus. The cognitive‐energetic model (Sergeant et al., 1999) specifies that the efficiency and effectiveness of one's cognitive functioning are related to both internal and external factors. These factors combine to create a situational state. Internal factors include one's tiredness and arousal. Arousal is of particular interest in the cognitive‐energetic model, as an optimum level of arousal has been related to increased task focus (Sergeant et al., 1999).

Combining valence and arousal, we arrive once more at the circumplex model of affect (Russel, 1980). Emotions from each quadrant of the circumplex model can influence classroom behaviour in different ways (Hospel et al., 2016). Especially, the combination of arousal and positivity has been found favourable for promoting on‐task behaviour and academic work more widely. For example, research has demonstrated that adolescents who report higher positive affect and more feelings of being energetic are more likely to show productive behaviours during school work (Mouratidis et al., 2017).

Tiredness, on the other hand, has been shown to negatively relate to cognitive task performance. In the context of cognitive functions, tiredness can be described as a state in which increased effort or motivation is required to maintain efficiency and ability (Ishii et al., 2014). When a person is in a state of tiredness rather than arousal during their cognitive task, they have two options: (1) they can maintain their level of cognitive output from rested states, by exerting more effort, or (2) they can reduce their level of output and maintain the same level of effort (Hockey, 1997). As tiredness is a signal from the body intended to make us seek rest and recovery (Ishii et al., 2014), the first option will not be sustainable. Reduced output is evident in many studies, and selective attention has been found to be particularly vulnerable to the effects of tiredness (Ishii et al., 2014).

Thus, it appears that fluctuations in affect and tiredness may influence task‐related behaviour differently. As affective states and tiredness fluctuate within persons, and even within the same day (Scrimin & Mason, 2015; Tetzlaff et al., 2021), we sought to investigate how pupils' affective states and tiredness fluctuate during an afternoon at school, if they are affected by PA and if they affect task‐related behaviour.

### Aims of this study

In order to expand previous studies, we investigated the dose–response relationship between acute PA intensity during PE lessons (dose) and task‐related behaviour in the classroom after PE (response), and whether learning experiences (affect, tiredness) mediated the associations between PA and task behaviour, as represented in Figure 1.

We posed two research questions:

1. Does PA predict post‐PE task behaviour?
2. Do (a) positive affect, (b) negative affect and (c) tiredness mediate between PA and task behaviour?

We hypothesized that (1) MPA and VPA increase on‐task behaviour and reduce active off‐task behaviour, in line with Ma et al. (2014). With regard to the mediation effects (2), we hypothesized that (2a) PA, and particularly MVPA, would increase positive affect, based on results from Bourke et al. (2021) and Dunton et al. (2014). Further, based on the cognitive‐energetic model (Sergeant et al., 1999), we expected mediated effects of PA on behaviour via positive affect; the increased positive affect after MVPA was hypothesized to lead to more on‐task behaviour. For negative affect (2b), we expected that negative affect would be reduced after MVPA (Dunton et al., 2014), and that this reduced negative affect would lead to more on‐task behaviour. For tiredness, also based on the cognitive‐energetic model (Sergeant et al., 1999) and results from Dunton et al. (2014), we expected (2c) MPA to decrease tiredness, and a mediated effect of MPA via decreased experienced tiredness, to more on‐task behaviour. Finally, (2c) we expected a mediated path from VPA via tiredness to on‐task and passive off‐task behaviours, whereby VPA would increase tiredness in the classroom after PA and lead to more passive off‐task behaviour and less on‐task behaviour (Hockey, 1997).

## METHOD

### Sample and procedure

Ethical approval for this study was granted by the Departmental Research Ethics Committee at the Oxford University Department for Education. In total, 101 children from eight classrooms (grades 3 to 5) of four primary schools in Oxfordshire, South‐East England, took part in this investigation. Children were eligible to participate if they could fully take part in PE lessons and were not diagnosed with behavioural or neurological impairments. Parents provided informed consent for their child, and the child provided written assent to participate. All children with consent completed questionnaires about their learning experiences, and up to 12 children per classroom wore accelerometers and were observed. If more than 12 children in one classroom had parental consent, the class teacher selected 12 students to ensure an even distribution of sex and academic achievement levels. Thus, data from 78 children (*M*_age_ = 9.3 years, *SD* = .6 years; 43 females) were analysed. Children were weighed using digital scales (Salter, model 9018S‐SV‐3R) and measured with a measuring tape affixed to the wall. Body mass index (BMI) values were calculated and World Health Organization (WHO) parameters for age‐ and sex‐adjusted BMI z‐scores were applied (*M* = .58, range = −1.77 to 3.71). Sample descriptives can be found in Table 1.

**TABLE 1:** Sample descriptives

Variable	Categories	*n*	*M*/%	*SD*	Min	Max	*n*_*ti*_
Age		78	9.23	.64	7.90	10.44	
Weight (kg)		78	33.88	9.85	18.50	73.90	
Height (m)		78	1.36	.08	1.13	1.56	
BMI z‐score^a^		78	.58	1.31	−1.77	3.71	
PE lessons^b^		78	5.51	.82	2	6	436
Self‐reports^c^		78	36.57	6.36	12	42	2811
Regularly active^d^	Yes	48	61.5%				
	No	30	38.5%				
Sex	Girl	43	55.1%				
	Boy	35	44.9%				
Year group	Y3	12	15.4%				
	Y4	41	52.5%				
	Y5	25	32.1%				

^a^ BMI z‐scores for age and sex, using WHO guidelines.

^b^ The number of PE lessons each child took part in.

^c^ The number of self‐reports each participant completed. Self‐reports included five situation‐specific constructs: task enjoyment, task difficulty, tiredness, positive and negative affect.

^d^ Based on objectively measured moderate‐to‐vigorous physical activity and UK government recommendations for children's activity levels.

The intervention consisted of six sessions (one per condition), lasting one afternoon each and taking place once per week for six weeks. The PE lessons were delivered by the lead researcher to ensure maximum consistency across classrooms. Within each class, the intervention took place on the same day of the week, and across all classes, each session lasted from the start of the afternoon lessons after lunch until the end of the school day. On the day before the intervention session, the research team fitted each participant with a GENEActiv accelerometer (Activinsights, 2012) on the wrist of their nondominant hand. The device was fitted just before children went home from school and was worn for 24 h. Before and after each PE lesson, participants took part in ‘business as usual’ classroom lessons, delivered by their usual class teacher. During classroom lessons, up to 12 participants' task behaviour was observed using a momentary time sampling protocol. At the start, middle and end of each observation period, and at the end of the PE lesson, participants rated their learning experiences on tablets with a purpose‐built app.

### Physical activity

The six PE lessons in this intervention followed a 3 (intensity: low, medium, or high) by 2 (complexity: low or high) within‐person design. PE lessons were executed in random order for each class. Lessons were designed to comply with the requirements of the UK National Curriculum for PE and no specialist equipment was required. Two lessons targeted vigorous physical activity (VPA), two were aimed at the moderate physical activity (MPA) and two were at the light physical activity (LPA). More detailed descriptions of each PE lesson were published previously (Heemskerk et al., 2019).

Accelerometry data, in 1‐second epochs, was processed with GGIR in R (Migueles et al., 2019). We extracted the duration participants were sedentary, and engaged in light, moderate and vigorous activity for the 24 h period during which the device was worn. Cut‐points for light, moderate and vigorous PA from Hildebrand et al. (2014) for wrist‐worn GENEActiv monitors were applied. Files with a minimum of 8 h of valid data during the daytime (7 am until 8 pm) were included for analyses of free‐living PA. For PE lessons, at least 30 min of valid data were required for inclusion. As a benchmark for ‘regularly active children’, we used UK government guidelines for PA: 60 min of moderate‐to‐vigorous physical activity (MVPA) per day (Chief Medical Officers, 2019). Children who met this guideline on at least 75% of recorded days were classed as ‘regularly active children’ (61.5% of the sample). PA during PE lessons was excluded from free‐living PA analyses due to the intervention manipulation having a potential impact on the values.

### Learning experiences

Participants reported a range of situation‐specific subjective learning experiences (task enjoyment, perceived task difficulty, tiredness, positive and negative emotions) on 6‐point Likert scales, three times per classroom lesson and once after the PE lesson. The questionnaire included 11 items illustrated with emoji symbols (Rane, 2017) with responses from zero to five stars on the touch screen of a hand‐held tablet. In the context of this paper, only the results of affect and tiredness are presented. In the classroom, tiredness was measured by one item (‘Are you tired?’), whereas affect was measured with eight items (four positive, four negative), using the question ‘How do you feel right now?’ After PE, the question ‘Are you tired?’ was replaced with an item to measure their perceived intensity of the PE lesson (rate of perceived exertion, RPE: ‘How tiring was your PE lesson?’). A total of 2811 self‐reports were completed by participants, of which 436 related to PE lessons. On average, each participant completed 5.5 PE questionnaires and 30.4 classroom questionnaires (Table 2).

**TABLE 2:** Descritive statistics of behaviour and self‐reports

Variable	Classroom before PE				PE lesson				Classroom after PE			
	*N*	*M* (*SD*)	Min	Max	*N*	*M* (*SD*)	Min	Max	*N*	*M* (*SD*)	Min	Max
On‐task	15,305	72.4 (20.8)	10.0	100.0					16,827	76.0 (14.9)	28.0	100.0
Passive off‐task	2198	10.5 (10.8)	0.0	64.0					1836	8.2 (7.5)	0.0	38.0
Active off‐task	2912	13.8 (15.5)	0.0	80.0					2952	13.4 (12.5)	0.0	70.0
Tiredness/RPE^a^	1252	2.76 (1.47)	0	5	451	3.30 (1.51)	0	5	1259	3.13 (1.54)	0	5
Excited	1176	3.69 (1.57)	0	5	416	3.48 (1.67)	0	5	1139	3.33 (1.70)	0	5
Happy	1174	3.99 (1.37)	0	5	414	3.94 (1.41)	0	5	1141	3.75 (1.50)	0	5
Calm	1170	3.87 (1.40)	0	5	415	3.53 (1.54)	0	5	1147	3.76 (1.47)	0	5
Relaxed	1165	3.89 (1.42)	0	5	408	3.57 (1.57)	0	5	1139	3.71 (1.49)	0	5
Bored	1109	2.23 (1.51)	0	5	394	2.24 (1.57)	1	5	1147	2.62 (1.65)	0	5
Sad	1040	1.58 (1.17)	0	5	381	1.69 (1.30)	0	5	1077	1.71 (1.33)	0	5
Angry	1050	1.57 (1.22)	0	5	383	1.74 (1.34)	1	5	1063	1.71 (1.35)	0	5
Nervous	1067	1.56 (1.15)	0	5	386	1.61 (1.24)	0	5	1093	1.70 (1.32)	0	5

^a^ RPE = rate of perceived exertion; at the end of PE lessons, participants answered the question ‘How tiring was your PE lesson?’

To test the structural validity of the emotion‐constructs in our analytic sample, we carried out a multilevel confirmatory factor analysis, with situations (t) nested in days (d), nested in children (i), i.e. in long data. Good model fit was determined by a Comparative Fit Index (CFI) >.95, a root mean square error (RMSEA) <.05 and level‐specific standardized root mean square residuals (SRMR) <.05. The a priori four‐factor model (positive activating, positive deactivating, negative activating, negative deactivating) did not fit the data, and upon closer inspection the items ‘bored’ and ‘excited' were removed. The resulting two‐factor solution (positive and negative affect) fitted the data well (*n*_*i*_ = 78, *n*_*di*_ = 372, *n*_*tdi*_ = 2630; $\chi_{28}^{2}$ = 30.29, *p* = .34; RMSEA = .006; CFI = .996; SRMR_*t*_ = .017; SRMR_*d*_ = .049; SRMR_*i*_ = .024), with three positive emotions, ‘happy’, ‘calm’, ‘relaxed’ and three negative emotions ‘sad’, ‘angry’, ‘nervous’, loading on their respective factors (Table 3).

**TABLE 3:** Confirmatory factor analysis results

Factor	Item	Factor loading		
		Within	Between weeks	Between persons
Positive affect	Happy	.65	.82	.66
	Calm	.79	.98	1.00
	Relaxed	.82	.97	.82
Negative affect	Sad	.80	.85	.99
	Angry	.83	.90	.93
	Nervous	.60	.53	.87

*Note*: Reported factor loadings are standardized results. The within level represents time points within the intervention afternoon. Time points were nested within weeks, as intervention afternoon occurred per week. At the highest level, weeks were nested within participants.

For our main analyses, we aggregated the three reports (start, middle and end of lesson) within each pre‐ and post‐PE classroom lesson, so that for each lesson three variables were created: a single‐item construct (tiredness) and two 3‐item constructs (positive affect, negative affect). PE self‐reports were completed once per lesson, and again, three variables were created: tiredness (single‐item construct), and positive and negative affect (two 3‐item constructs).

### Task behaviour observations

Observations of the task behaviour of the target participants were made every 30 seconds for 25 min (six target children for each of two trained observers) into one of four categories (*N*_observations_ = 43,299). A rating of ‘on‐task’ was given if the participant displayed goal‐directed behaviours for completing the task set by the teacher. In the absence of goal‐directed behaviours, ‘off‐task passive’ was recorded if they instead were inactive (e.g. staring, daydreaming), or ‘off‐task active’ if the child was active (e.g. non‐task‐related talking, moving about in their seat or around the classroom). Finally, if the behaviour could not be categorized as one of those three (e.g. gone to the toilet, another person obstructing the view of the observer), the time interval was rated ‘other’. Inter‐rater reliability for the two researchers carrying out the observations was determined based on 30 min of double‐coded observations (360 observations) and found to be good (Cohen's Kappa = .80).

It has been found that on‐task behaviour varies between instructional activities (Godwin et al., 2013; Heemskerk & Malmberg, 2020). Thus, alongside the participant's behaviour, the type of task set by the teacher was noted (teacher‐led whole‐class instruction, teacher one‐to‐one support, independent work, partner work, small group work, test, or other). For the purpose of the analyses, observations where behaviour and tasks were concurrently coded ‘other’ were removed, and only lessons with a minimum of 40 valid observations were included. Behaviour data were aggregated per behaviour type to create one rating of the proportion of each behaviour in the pre‐PE lesson and post‐PE lesson.

### Analytic procedures

The resulting data set contained one line of data per day per child, i.e. 372 days, nested in 78 children (wide data), with variables for the pre‐PE lesson (behaviour and self‐report) as baseline measures for that day, for the PE lesson (PA measures and self‐report), and for the post‐PE lesson (behaviour and self‐report) as the outcome and mediator variables for that day. We specified a series of multilevel structural equation models in Mplus8 (Muthen & Muthen, 2017). Throughout this paper, we report standardized coefficients, along with standard errors and *p*‐values for significance. For significant paths (*p* < .05), effect sizes were calculated following Tymms (2004), with ES < .2 = negligible, ≥.2 = small, ≥.5 = medium and ≥.8 = large. In the figures of path models, all nonsignificant paths have been omitted for the clarity of the graphs.

**FIGURE 2:**
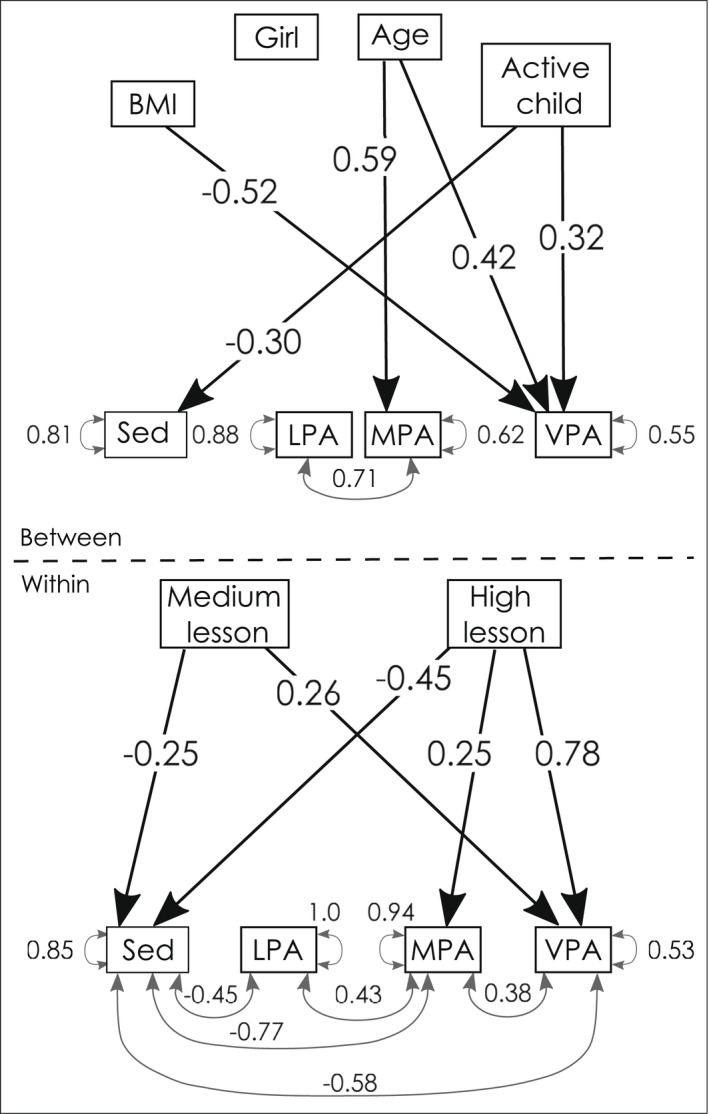
Two‐level model of the determinants of recorded time at physical activity intensities during physical education lessons. *Note*: LPA, light physical activity; MPA, moderate physical activity; Sed, sedentary behaviour; VPA, vigorous physical activity; BMI was z‐scored following WHO norms (De Onis et al., 2007), age in fractions of years, active child = achieved a minimum of 60 min moderate‐to‐vigorous physical activity for 75% of the six study‐days. There were three intervention conditions, low‐, medium‐ and high‐intensity PA physical education (PE) lessons. In the model, we use low‐PE as comparison group

In a preliminary model, we inspected the variance components of our key independent variable (PA), by partitioning the variance into within and between parts. Intraclass correlations (ICC) demonstrated that sedentary behaviour ($\rho_{\mathrm{ICC}}$ = .24), light PA (LPA) ($\rho_{\mathrm{ICC}}$ = .49), MPA ($\rho_{\mathrm{ICC}}$ = .08) and VPA ($\rho_{\mathrm{ICC}}$ = .05) had substantive within‐child variance across the six PE lessons. We then, as a manipulation check, included lesson‐type as two dummy‐coded predictors of PE at the within level (medium and high intensity of PE, using low‐intensity PE as baseline). Relative to low‐intensity lessons, pooled within‐level effects showed that during medium‐intensity lessons, children were less sedentary and recorded more VPA. During high‐intensity lessons, they were less sedentary and recorded more moderate and vigorous PA, see Table 4 and Figure 2 (within level).

**TABLE 4:** Determinants of recorded time at physical activity intensities during physical education lessons

Level	Outcome	Predictor	β	*SE*	*p*	ES^b^
Between	Sedentary time	Regularly active^a^	−.30	.124	.017*	−.61
Within	Sedentary time	Medium lesson	−.25	.102	.013*	−.11
	Sedentary time	High lesson	−.45	.122	<.001***	−.19
Between	MPA^c^	Age	.59	.239	.013*	1.19
Within	MPA	High lesson	.25	.083	.003**	.17
Between	VPA^d^	BMI z‐score^e^	−.52	.183	.005**	−1.04
	VPA	Regularly active	.32	.111	.005**	.65
	VPA	Age	.42	.103	<.001***	.84
Within	VPA	Medium lesson	.26	.069	<.001***	.22
	VPA	High lesson	.78	.082	<.001***	.67

^a^ Based on objectively measured moderate‐to‐vigorous physical activity and UK government recommendations for children's activity levels.

^b^ Effect size: <.2 = negligible, ≥.2 = small, ≥.5 = medium and ≥.8 = large.

^c^ Moderate physical activity.

^d^ Vigorous physical activity.

^e^ BMI z‐scores for age and sex, using WHO guidelines.

*p ≤ .05; **p ≤ .01; ***p ≤ .001.

Next, at the between‐level of the model, we inspected individual differences in PA according to individual characteristics (age, sex, BMI and habitual PA) and contextual factors (classroom and school). Children with higher BMI z‐score recorded less VPA, whereas regularly active children recorded more VPA, and less sedentary time. Older children recorded more MPA and VPA (Table 4 and Figure 2, between‐level). As we found differences between the eight classrooms ($\rho_{\mathrm{ICC}}$ = .07 to $\rho_{\mathrm{ICC}}$ = .42), we subsequently adjusted the standard errors of parameter estimates for differences between classrooms, using the COMPLEX command in Mplus, to reduce the risk of type I errors.

To answer the first research question, we specified models in which we regressed post‐PE task behaviour on PA, controlling for pre‐PE task behaviour and covariate effects at the between‐level, for each type of task behaviour separately (on‐task, active off‐task, passive off‐task). For interpreting the dose–response effects of PA on each child's task behaviour we centered pre‐PE task behaviour within children.

To answer the second research question, we included positive affect, negative affect and tiredness as mediators between PA and task behaviour. We controlled for effects of child characteristics at the between‐level.

## RESULTS

### Does PA predict task‐related behaviour?

Participants were on‐task on average 72.4% of the observed pre‐PE lessons (*SD* = 20.8) and 76.0% of the post‐PE lessons (*SD* = 14.9). Before and after PE, active off‐task behaviour was more prevalent than passive (Table 2). Children with a higher BMI z‐score were more passively off‐task. Girls were less actively off‐task than boys and more on‐task.

**FIGURE 3:**
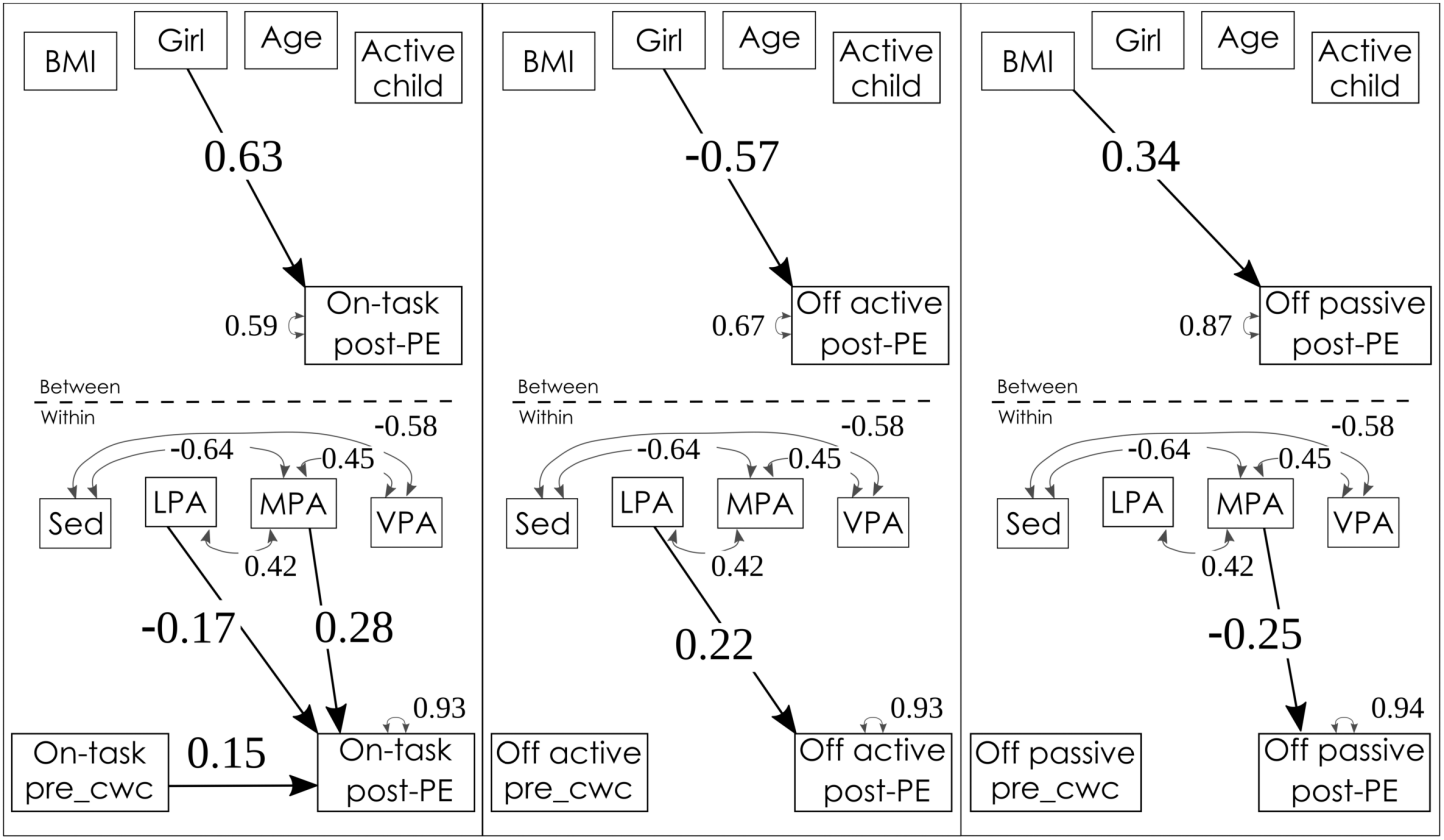
Physical activity and task behaviour. *Note*: _cwc, centered within cluster; LPA, light physical activity; MPA, moderate physical activity; Sed, sedentary behaviour; VPA, vigorous physical activity; BMI was z‐scored following WHO norms (De Onis et al., 2007), age in fractions of years, active child = achieved a minimum of 60 min moderate‐to‐vigorous physical activity for 75% of the six study‐days

Our first hypothesis was that moderate (MPA) and vigorous physical activity (VPA) would increase on‐task behaviour and reduce active off‐task behaviour, in line with Ma et al. (2014). Partially consistent with this hypothesis, a longer duration of MPA, but not VPA, predicted an increase in on‐task behaviour and decreased passive off‐task behaviours. Light physical activity (LPA) predicted less on‐task behaviour in the classroom after the PE lesson and more active off‐task behaviour (Table 5 and Figure 3, within‐level).

**TABLE 5:** Physical activity and task behaviour after physical education

Level	Outcome	Predictor	β	*SE*	*p*	ES^a^
On‐task behaviour						
Between	On‐task	Sex^b^	.62	.097	<.001***	1.19
Within	On‐task	On‐task before PE	.15	.050	<.001***	.28
	On‐task	LPA^c^	−.17	.065	.010*	−.31
	On‐task	MPA^d^	.28	.057	<.001***	.57
Active off‐task behaviour						
Between	Off active	Sex	−.50	.143	<.001***	−1.06
Within	Off active	LPA	.22	.074	.003**	.43
Passive off‐task behaviour						
Between	Off passive	BMI z‐score^e^	.29	.096	.001**	.83
Within	Off passive	MPA	−.25	.078	.001**	−.52

^a^ Effect size: <.2 = negligible, ≥.2 = small, ≥.5 = medium and ≥.8 = large.

^b^ 0 = boy, 1 = girl.

^c^ Light physical activity.

^d^ Moderate physical activity.

^e^ BMI z‐scores for age and sex, using WHO guidelines.

*p ≤ .05; **p ≤ .01; ***p ≤ .001.

### Do learning experiences mediate the effects of PA on‐task‐related behaviour?

**FIGURE 4:**
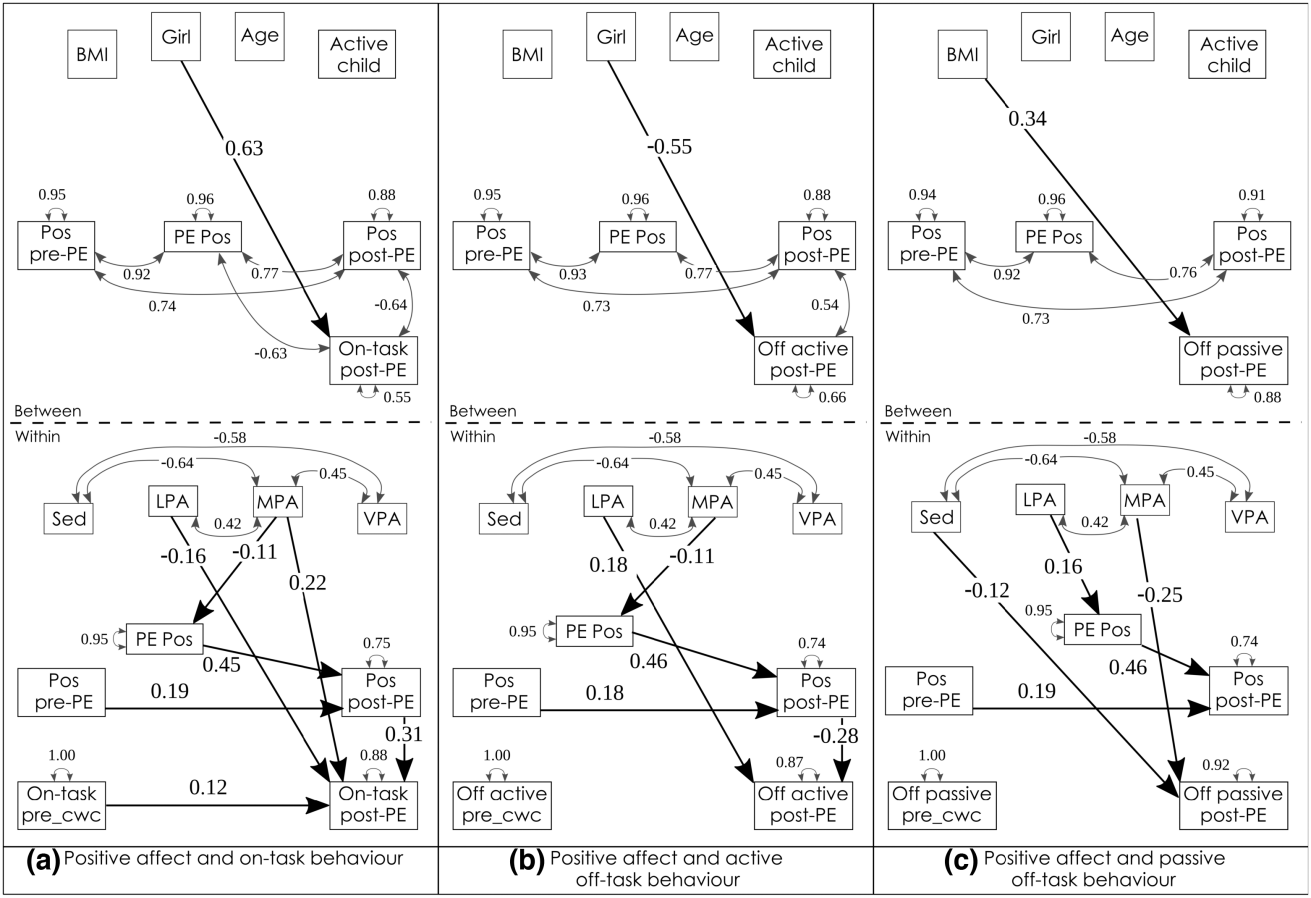
Physical activity, positive affect and task behaviour. *Note*: _cwc, centered within cluster; LPA, light physical activity; MPA, moderate physical activity; Pos, positive affect; Sed, sedentary behaviour; VPA, vigorous physical activity; BMI was z‐scored following WHO norms (De Onis et al., 2007), age in fractions of years, active child = achieved a minimum of 60 min moderate‐to‐vigorous physical activity for 75% of the six study‐days. Model fit: (a) RMSEA = .084, CFI = .894, SRMR = .068/.017; (b) RMSEA = .076, CFI = .908, SRMR = .072/.020; (c) RMSEA = .064, CFI = .934, SRMR = .063/.020

Positive and negative affect were unaffected by sex, BMI z‐score, age, or regular MVPA (Figures 4 & 6, between‐level). Regularly active children reported less tiredness throughout the afternoon. Their perceived rate of exertion (RPE) during the PE lesson was lower in regularly active children and in older children. No other characteristics had a significant impact on self‐reported tiredness or RPE (Table 8 and Figure 7, between‐level).

### Physical activity, positive affect and task‐related behaviour

With regard to the mediation effect, we hypothesized (2a) that PA, and particularly MVPA, would increase positive affect based on results from Bourke et al. (2021) and Dunton et al. (2014). Further, based on the cognitive‐energetic model (Sergeant et al., 1999), we expected mediated effects of PA on behaviour via positive affect. The increased positive affect after MVPA was hypothesized to lead to more on‐task behaviour.

**FIGURE 5:**
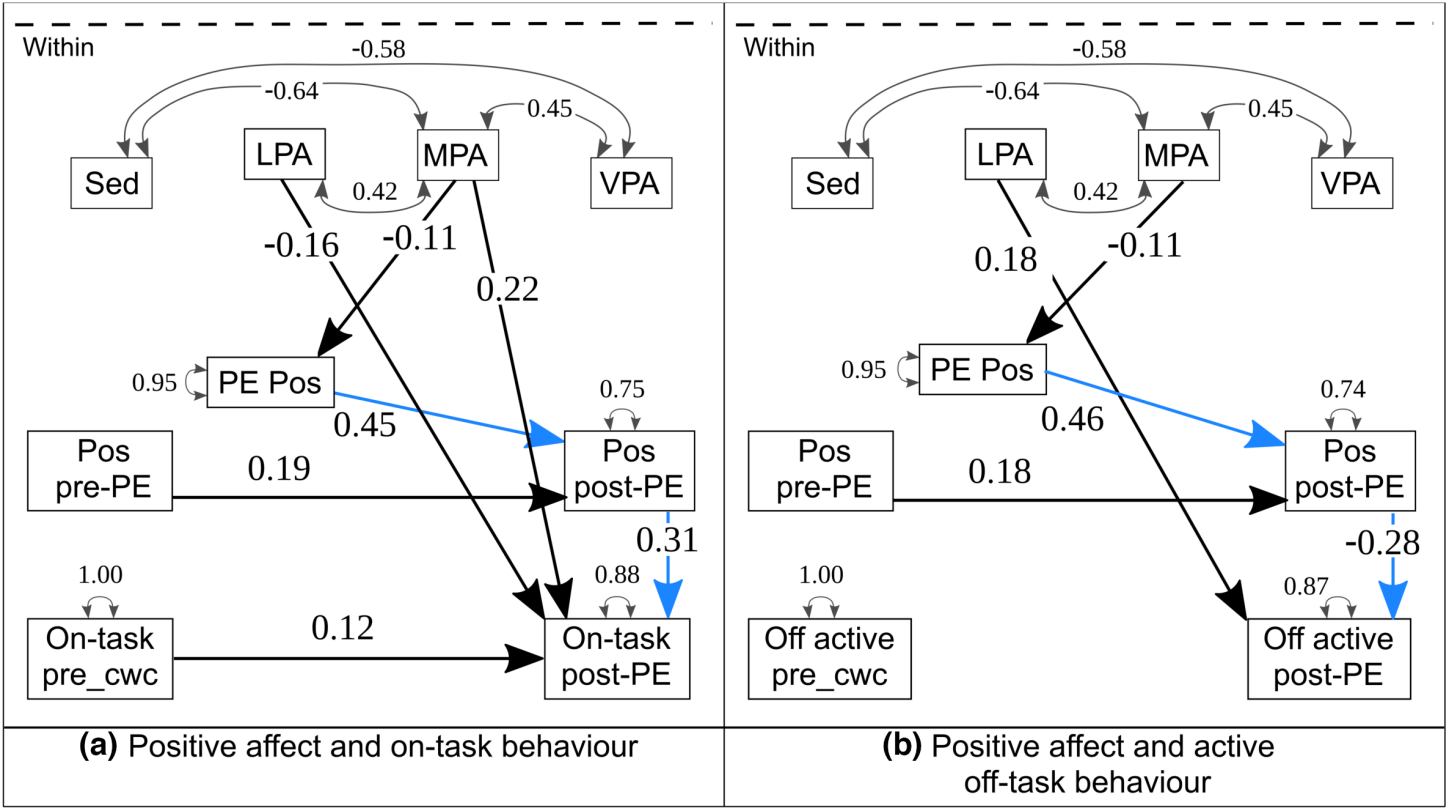
Indirect paths. *Note*: Indirect paths in blue ink. Model (a) β = .14; model (b) β = −.13. _cwc, centered within cluster; LPA, light physical activity; MPA, moderate physical activity; Pos, positive affect; Sed, sedentary behaviour; VPA, vigorous physical activity. Model fit: As in Figure 4

In contrast to our hypothesis, positive affect during PE was greater when children recorded more LPA during the PE lesson. More MPA was related to less positive affect during PE. Higher positive affect ratings during PE also predicted greater positive affect after PE. The hypothesized path from positive affect to task‐related behaviour was confirmed: positive affect in the classroom predicted more on‐task behaviour and less active off‐task behaviour after PE (Table 6 and Figure 4). Indirectly, more positive affect during PE led to more on‐task and less active off‐task behaviour after PE (β = .14, *SE* = .061, *p* = .023, ES = .05 and β = − .13, *SE* = .056, *p* = .020, ES = −.03, respectively, see blue paths in Figure 5).

**TABLE 6:** Physical activity, positive affect and task‐related behaviour after physical education

Level	Outcome	Predictor	β	*SE*	*p*	ES^a^
On‐task behaviour						
Between	On‐task	Sex^b^	.63	.083	<.001***	1.20
Within	Pos affect post‐PE	Pos affect pre‐PE	.19	.063	.003**	.37
	Pos affect post‐PE	Pos affect PE	.45	.115	<.001***	.90
	Pos affect PE	MPA^c^	−.11	.053	.031*	−.23
	On‐task	On‐task before PE	.12	.050	.013**	.25
	On‐task	LPA^d^	−.16	.053	.003**	−.32
	On‐task	MPA	.22	.061	<.001***	.43
	On‐task	Pos affect post‐PE	.31	.105	.003**	.60
Active off‐task behaviour^e^						
Between	Off active	Sex	−.55	.127	<.001***	−1.12
Within	Pos affect post‐PE	Pos affect pre‐PE	.18	.059	.002**	.37
	Pos affect post‐PE	Pos affect PE	.46	.112	<.001***	.90
	Off active	LPA	.18	.072	.011**	.35
	Off active	Pos affect post‐PE	−.28	.100	.005**	−.56
Passive off‐task behaviour^e^						
Between	Off passive	BMI z‐score^f^	.34	.095	<.001***	.83
Within	Pos affect PE	LPA	.16	.078	.043*	.32
	Off passive	MPA	−.25	.063	<.001***	−.52
	Off passive	Sedentary	−.12	.055	.026*	−.18

^a^ Effect size: <.2 = negligible, ≥.2 = small, ≥.5 = medium and ≥.8 = large.

^b^ 0 = boy, 1 = girl.

^c^ Moderate physical activity.

^d^ Light physical activity.

^e^ Parameters with the same values as in the models for other behaviours are not reported again.

^f^ BMI z‐scores for age and sex, using WHO guidelines.

*p ≤ .05; **p ≤ .01; *p* ≤ .001.

#### Physical activity, negative affect and task‐related behaviour

**FIGURE 6:**
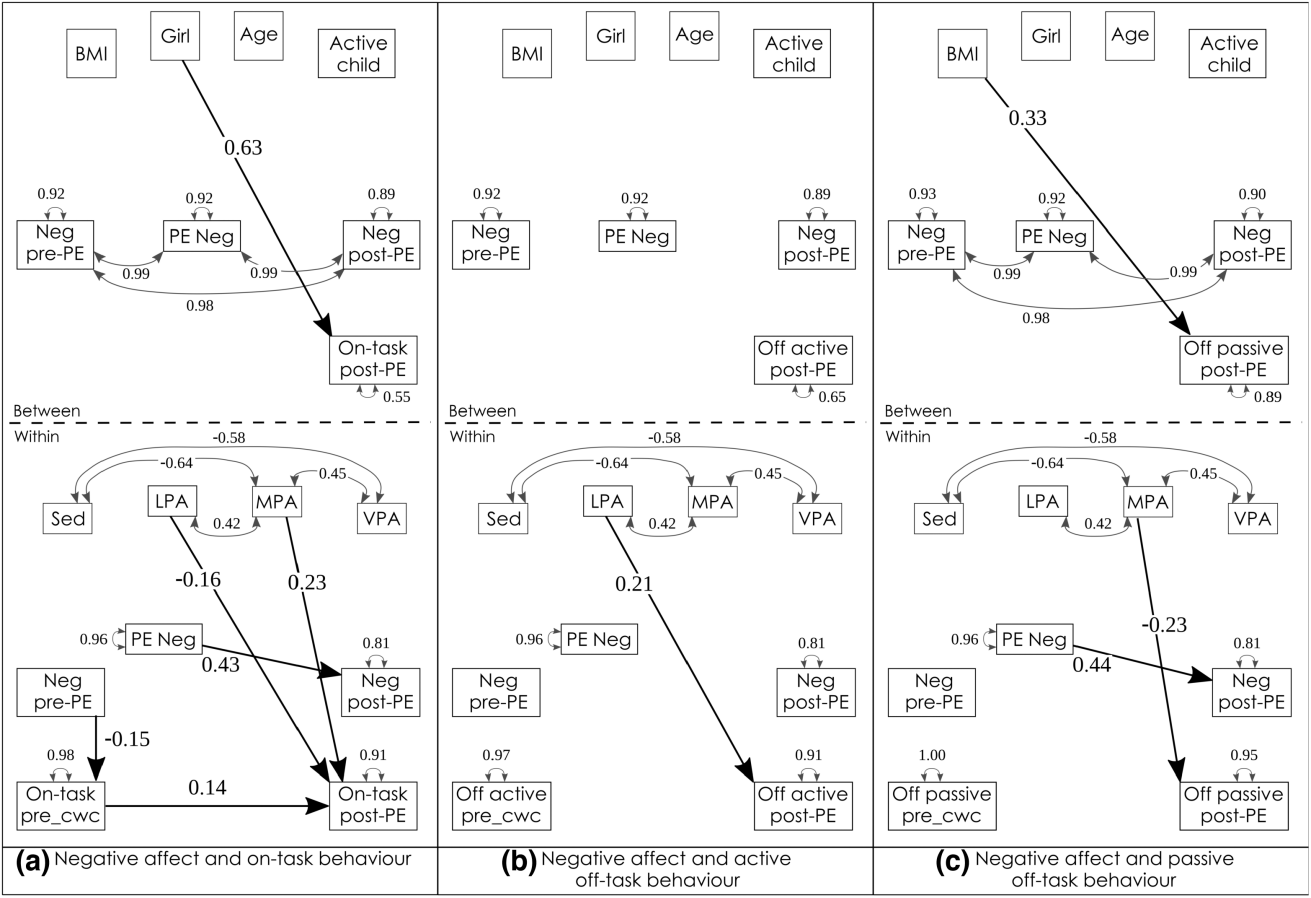
Physical activity, negative affect and task behaviour. *Note*: _cwc, centered within cluster; LPA, light physical activity; MPA, moderate physical activity; Neg, negative affect; Sed, sedentary behaviour; VPA, vigorous physical activity; BMI was z‐scored following WHO norms (De Onis et al., 2007), age in fractions of years, active child = achieved a minimum of 60 min moderate‐to‐vigorous physical activity for 75% of the six study‐days. Model fit: (a) RMSEA = .086, CFI = .898, SRMR = .039/.007; (b) RMSEA = .056, CFI = .952, SRMR = .054/.007; (c) RMSEA = .034, CFI = .983, SRMR = .038/.005

For negative affect (hypothesis 2b), we expected that negative affect would be reduced after MVPA (Dunton et al., 2014), and that this reduced negative affect would lead to more on‐task behaviour. Our hypothesis was not confirmed, as negative affect after PE did not relate to task‐related behaviour, thus no mediated effects of PA via negative affect on task‐related behaviour were found (Table 7 and Figure 6). However, children who felt more negative affect during PE also reported higher negative affect once they were back in the classroom after PE.

**TABLE 7:** Physical activity, negative affect and task‐related behaviour after physical education

Level	Outcome	Predictor	β	*SE*	*p*	ES^a^
On‐task behaviour						
Between	On‐task	Sex^b^	.63	.089	<.001***	1.20
Within	Neg affect post‐PE	Neg affect PE	.43	.033	<.001***	.87
	On‐task pre‐PE	Neg affect pre‐PE	−.15	.060	.013*	−.30
	On‐task	On‐task before PE	.14	.054	.012*	.27
	On‐task	LPA^c^	−.16	.056	.005**	−.32
	On‐task	MPA^d^	.23	.059	<.001***	.43
Active off‐task behaviour^e^						
Within	Off active	LPA	.21	.071	.004**	.40
Passive off‐task behaviour^e^						
Between	Off passive	BMI z‐score^f^	.33	.095	.001**	.74
Within	Neg affect post‐PE	Neg affect PE	.44	.041	<.001***	.88
	Off passive	MPA	−.23	.064	<.001***	−.52

^a^ Effect size: <.2 = negligible, ≥.2 = small, ≥.5 = medium and ≥.8 = large.

^b^ 0 = boy, 1 = girl.

^c^ Light physical activity.

^d^ Moderate physical activity.

^e^ Parameters with the same values as in the models for other behaviours are not reported again.

^f^ BMI z‐scores for age and sex, using WHO guidelines.

*p ≤ .05; **p ≤ .01; ***p ≤ .001.

#### Physical activity, tiredness and task‐related behaviour

For tiredness, also based on the cognitive‐energetic model (Sergeant et al., 1999) and results from Dunton et al. (2014), we expected MPA to decrease tiredness, and a mediated effect of MPA via decreased experienced tiredness, to more on‐task behaviour (hypothesis 2c). Finally, we expected a mediated path from VPA via tiredness to on‐task and passive off‐task behaviours, whereby VPA would increase tiredness in the classroom after PA and lead to more passive off‐task behaviour and less on‐task behaviour (Hockey, 1997).

**FIGURE 7:**
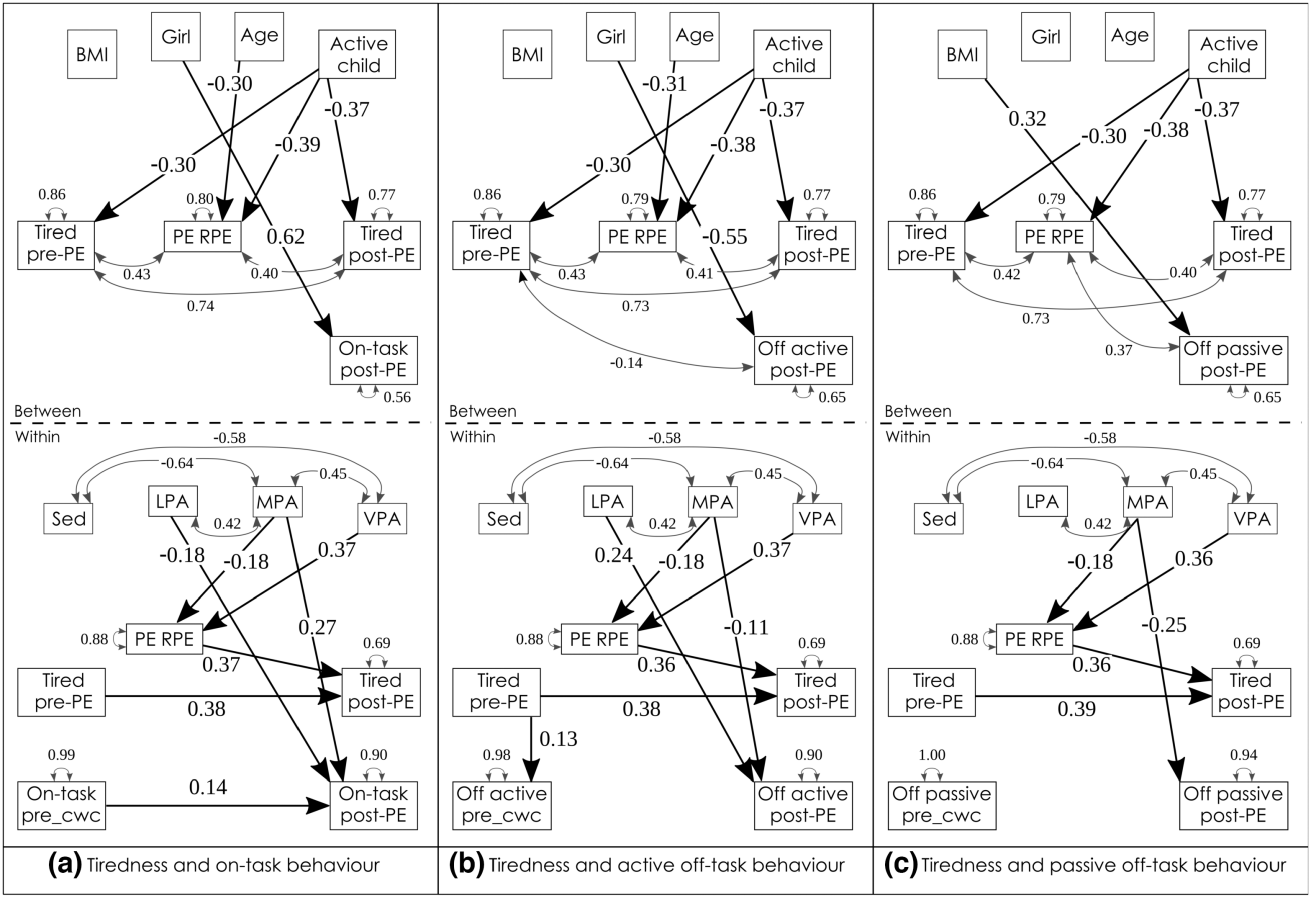
Physical activity, tiredness and task behaviour. *Note*: _cwc, centered within cluster; RPE, rate of perceived exertion; LPA, light physical activity; MPA, moderate physical activity; Tired, tiredness in the classroom; Sed, sedentary behaviour; VPA, vigorous physical activity. BMI was z‐scored following WHO norms (De Onis et al., 2007), age in fractions of years, active child = achieved a minimum of 60 min moderate‐to‐vigorous physical activity for 75% of the six study‐days. Model fit: (a) RMSEA = .024, CFI = .992, SRMR = .036/.008; (b) RMSEA = .059, CFI = .946, SRMR = .050/.010; (c) RMSEA = .000, CFI = 1.00, SRMR = .029/.006

Whereas VPA increased participants' rate of perceived exertion, MPA decreased their RPE. PE lessons with a higher RPE led to greater tiredness afterwards. Higher ratings of tiredness in the classroom did not affect task‐related behaviour after PE (Table 8 and Figure 7, within‐level). No mediated effects of PA on‐task‐related behaviour via tiredness were found, as the path from tiredness post‐PE to task‐related behaviour was not significant. In line with our hypothesis (2c), children did indirectly feel less tired after PE with high‐MPA content (β = − .07, *SE* = .020, *p* = .001, ES = −.01) and more tired after PE with high‐VPA content (β = .13, *SE* = .036, *p* < .001, ES = .03).

**TABLE 8:** Physical activity, tiredness and task‐related behaviour after physical education

Level	Outcome	Predictor	β	*SE*	*p*	ES^a^
On‐task behaviour						
Between	Tired pre‐PE	Regularly active^b^	−.30	.072	<.001***	−.61
	Tired post‐PE	Regularly active	−.37	.124	.003**	−.74
	RPE^c^	Regularly active	−.39	.133	.004**	−.78
	RPE	Age	−.30	.154	.049*	−.61
	On‐task	Sex^d^	.62	.083	<.001***	1.19
Within	Tired post‐PE	Tired pre‐PE	.38	.075	<.001***	.75
	Tired post‐PE	RPE	.37	.069	<.001***	.74
	RPE	MPA^e^	−.18	.035	<.001***	−.37
	RPE	VPA^f^	.37	.052	<.001***	.73
	On‐task	On‐task before PE	.14	.046	.003**	.27
	On‐task	LPA^g^	−.18	.054	.001**	−.36
	On‐task	MPA	.27	.052	<.001***	.53
Active off‐task behaviour^h^						
Between	RPE	Regularly active	−.38	.138	.006**	−.78
	RPE	Age	−.31	.153	.045*	−.61
	Off active	Sex	−.55	.126	<.001***	−1.12
Within	Tired post‐PE	RPE	.36	.072	<.001***	.73
	Off active pre‐PE	Tired pre‐PE	.13	.038	<.001***	.25
	Off active	LPA	.24	.073	.001**	.50
	Off active	MPA	−.11	.045	.015*	−.24
Passive off‐task behaviour^h^						
Between	Off passive	BMI z‐score^i^	.32	.092	.001**	.74
Within	Tired post‐PE	Tired pre‐PE	.39	.073	<.001***	.77
	RPE	VPA	.36	.050	<.001***	.72
	Off passive	MPA	−.25	.062	<.001***	−.52

^a^ Effect size: <.2 = negligible, ≥.2 = small, ≥.5 = medium and ≥.8 = large.

^b^ Based on objectively measured moderate‐to‐vigorous physical activity and UK government recommendations for children's activity levels.

^c^ Rate of perceived exertion.

^d^ 0 = boy, 1 = girl.

^e^ Moderate physical activity.

^f^ Vigorous physical activity.

^g^ Light physical activity.

^h^ Parameters with the same values as in the models for other behaviours are not reported again.

^i^ BMI z‐scores for age and sex, using WHO guidelines.

*p ≤ .05; **p ≤ .01; ***p ≤ .001.

## DISCUSSION

We investigated the dose–response relationships between objectively measured PA during PE lessons, self‐reported affect and tiredness, and observed task‐related behaviour during classroom lessons. We analysed between‐person differences and within‐person variations in task‐related behaviour, objectively measured PA, and self‐reported tiredness and affect. At the within level, we investigated the direct effects of PA intensity on task‐related behaviour and mediated effects of PA intensity, via tiredness and affect, on task‐related behaviour.

### Between‐person results

At the between‐level, we identified effects of regular moderate‐to‐vigorous physical activity (MVPA) on the perception of tiredness in the classroom, and on perceived PE lesson intensity, as per hypothesis 2c. However, we found no effect of regular MVPA (active child category) on ratings of affect, thus hypotheses 2a and 2b were not supported. Although, based on Bourke et al. (2021), the lack of acute effects on negative affect was not entirely unexpected, we had still expected to find effects of chronic MVPA at the between‐level. This unexpected result may be related to the selection criteria for the ‘active’ subsample in this study; we chose guidelines from the Chief Medical Officers (2019) as our criteria, using a cumulative measure across each recorded 24‐hour window. Thus, there is no guarantee that the PA recorded by the ‘active’ subsample in our study was performed in sustained bouts, which may be necessary to achieve PA benefits for mental well‐being.

Another possible explanation for not identifying an effect of regular PA on affect is that, unlike the participants in studies by Strauss et al. (2001) or Chan et al. (2019), our measure of regular PA required a greater amount of PA (at least 60 min), compared with the regular participation in only 10 to 15 min suggested by Chan et al. (2019), or the analysis of all naturally occurring MVPA across 1 week (Strauss et al., 2001). Therefore, in our study, participants who may have participated in 10 to 15 min of PA, but not accumulated at least 60 min of MVPA across the day, or those who did not exercise at above the MVPA intensity cut‐off, will not have been included in the ‘regularly active’ subsample.

Another potential reason we did not find support for hypothesis 2b comes into play at both the between and within level, and relates to the measurement of affect. In contrast to findings in both adults (Chan et al., 2019) and children (Dunton et al., 2014), no chronic or acute effects of PA were found for negative affect in our study. This may be due to our young sample having a limited emotional vocabulary, or a reluctance to report negative affect (Rosen & Tesser, 1970).

### Direct effects of physical activity on task‐related behaviour

At the within‐person level, we found that the types of task behaviour we observed responded differently to PA. Firstly, the findings partly confirm hypothesis 1; that on‐task behaviour would be higher after moderate physical activity (MPA). Unexpectedly, we found that on‐task behaviour was lower after light physical activity (LPA). However, these two intensities of PA affected different types of off‐task behaviour; there was less passive off‐task behaviour after MPA and more active off‐task behaviour after LPA.

Based on the cognitive‐energetic model (Sergeant et al., 1999), the MPA content of the PE lesson may have increased physiological arousal in participants, lasting into the subsequent classroom lesson and reducing passivity. The decrease in on‐task behaviour after LPA may not indicate a negative effect as such, as a decrease in on task behaviour usually observed over time (Grieco et al., 2016; Ma et al., 2014). Though it may suggest that a minimum level of exertion is required to counter this decline in on‐task behaviour and the increase in active off‐task behaviour.

Our hypothesized direct effect of vigorous physical activity (VPA) on task behaviour was not confirmed, as VPA did not directly influence behaviour in our sample. In the following sections, the absence of an effect of VPA is discussed in more detail in relation to the indirect effects. The combination of the negative effect of LPA on on‐task behaviour, the positive effect of MPA and the absence of an effect of VPA, is suggestive of an inverted U‐shaped relationship between PA intensity and on‐task behaviour, with MPA being the optimal dose for stimulating on‐task behaviour, as previously described by Tomporowski (2003).

### Effects of physical activity on self‐reported affect and tiredness

We found effects of PA on experienced tiredness and positive affect, but not negative affect. At the end of PE lessons with a greater LPA content, participants reported higher levels of positive affect, whereas MPA led to lower positive affect and VPA did not influence positive affect, in contrast to our hypothesis (2a) and unlike the results reported by Dunton et al. (2014). The items included to measure positive affect may explain this difference. In our study, positive affect was measured with the items ‘happy’, ‘calm’ and ‘relaxed’, whereas Dunton et al. (2014) used ‘happy’ and ‘joyous’. It could therefore be that our measure of positive affect was positively related to LPA and negatively to MPA due to it capturing more deactivating positivity, rather than activating emotions. Although our questionnaire included two activating and two deactivating positive emotions, only three of the four items loaded significantly onto the positive affect construct, and we were unable to analyse activating and deactivating affect separately. Another possibility is that our sample was somewhat younger than that of Dunton et al. (2014) (7 to 10 years old vs. 9 to 13 years old) and, despite explanations at the start of the project, their emotional vocabulary may not have been sufficiently developed to capture the nuances of the range of activating and deactivating emotions.

We did not find effects of PA on negative affect, contrary to our hypothesis (2b). It thus appears that positive affect is more easily influenced by situational factors during PE lessons than negative affect. Similar findings were reported by Bourke et al. (2021): they found that positive affect was more responsive to acute PA than negative affect and suggested that rather than being influenced by acute PA, reduced negative affect may be a long‐term effect, related to an accumulation of PA‐induced increases in positive affect. This would not be detected with the acute measures applied in the current study.

Another reason for not having detected an effect of PA on negative affect could be a floor‐effect for negative affect: before PE, 60% of participants rated their negative affect as ≤ 1.22 out of 5. This low starting point makes it difficult to drop even lower. The low levels of negative affect reported could reflect that our sample generally felt very little negativity, or may be related to a reporting bias, whereby participants were reluctant to provide high ratings for negative emotions (Rosen & Tesser, 1970).

With regard to tiredness, we found that subjective ratings of PE lesson intensity influenced tiredness after PE lessons, but objective measures of LPA, MVPA and VPA did not. The duration of MPA and VPA during PE did influence the perceived PA intensity in the hypothesized direction (2c): children rated high‐MPA PE lessons as less exerting, and high‐VPA PE lessons as more exerting. This fits with the cognitive‐energetic theory that PA of sufficient intensity leads to arousal (Sergeant et al., 1999), perceived alertness and feelings of being energized, but also that high‐intensity PA can lead to a depletion of the body's energy stores (Hockey, 1997), inducing tiredness.

### Mediated effects of physical activity on task‐related behaviour

We found no evidence to support the hypothesized full mediation paths (2a, 2b and 2c). However, part of the hypothesized path for positive affect (2a) was confirmed. Positive affect during PE indirectly predicted on‐task behaviour. As the mediation path from MPA to task behaviour was not significant, but the path from positive affect to task behaviour was, it appears that, alongside the intensity of the physical activity, the positive affect experienced during the PE lesson is most beneficial to children's ability to subsequently focus on their school work.

We found no mediating effects of tiredness or negative affect on task behaviour after PE (hypothesis 2b), as the mediating variables did not significantly predict task behaviour after PE. In the case of negative affect, PA also did not predict affect, as discussed in the previous section. Considering the low levels of negative affect reported by the participants, it is possible we did not find effects of negative affect on task‐related behaviour, because reported negative affect was not sufficiently high to have a detectable effect.

Where tiredness is concerned (hypothesis 2c), there may have been compensation for the experienced tiredness in the form of additional effort being exerted to remain on‐task, as set out by Hockey (1997) and Ishii et al. (2014). This would mean that, although participants felt tired, their task behaviour did not reflect this. It therefore could be the case that we did not find significant effects of VPA on task‐related behaviour, as the PA‐induced tiredness may have prevented arousal‐related benefits, whilst compensatory effort to stay on‐task prevented any negative effects. This is in contrast to the findings by Ma et al. (2014), where VPA led to increased on‐task behaviour. In their intervention, Ma et al. (2014) used 4‐min activities, as part of a 10‐min classroom break. It can be expected that a full PE lesson with high‐VPA content would induce greater tiredness than a 4‐min exercise break. Thus, the VPA accrued by participants in the study by Ma et al. (2014) may have led to arousal and improved on‐task behaviour, whereas our sample additionally induced tiredness and their task‐related behaviour remained unchanged. Still, VPA did prevent the decline in on‐task behaviour, which is usually observed over time.

### Strengths and limitations

The strengths of this study are the intensive longitudinal data collected, and the investigation of direct effects of PA as well indirect and mediation paths. This brings together several strands of the research literature and moves towards uncovering the processes by which PA influences task behaviour. There were three limitations to this study. First, as the sample size was moderate, the findings would benefit from replication in larger samples. Moreover, as our participants were fairly young, their ability to report their emotional states may have been limited. Finally, it must be noted that the intervention sought to compare different intensities of PE lesson PA with each other. There was no classroom‐based control condition; instead, low‐intensity PE lessons were the within‐person control condition in this study. This was to ensure that the effects of PA found in our study would not be due to the break from the classroom provided by the PE lesson and should be interpreted accordingly.

### Implications

Teachers can make use of PA to increase pupils' on‐task behaviour and improve their subjective learning experiences. As active off‐task behaviours are not only an indicator that the off‐task child is disengaged from the learning process, but also disrupts others in the same classroom, teachers are keen to minimize these behaviours. Teachers can prevent active, disruptive off‐task behaviours after PA, and reduce passive off‐task behaviours by providing PA opportunities of moderate rather than light intensity. Moreover, they can increase their students' positive affect and on‐task behaviour and reduce off‐task behaviour, by creating positive and enjoyable PA experiences during school.

## CONCLUSION

PE lessons can increase on‐task behaviour and reduce both passive and active off‐task behaviours. Positive affect and tiredness are indirectly involved in the impact of PA on task‐related behaviour. The greatest benefits were found for moderate PA and for PE lessons, which left children feeling positive. Moreover, regular participation in moderate‐to‐vigorous PA leads children to feel less tired during school lessons.

## AUTHOR CONTRIBUTIONS

**Christina Hubertina Helena Maria Heemskerk:** Conceptualization; data curation; formal analysis; investigation; methodology; project administration; resources; visualization; writing – original draft; writing – review and editing. **Steve Strand:** Conceptualization; methodology; supervision. **Lars‐Erik Malmberg:** Conceptualization; data curation; formal analysis; funding acquisition; investigation; methodology; supervision; visualization; writing – original draft; writing – review and editing.

## CONFLICT OF INTEREST

All authors declare no conflict of interest.

## Data Availability

The data that support the findings are available from the corresponding author.
